# Relationship between sprint, jump, dynamic balance with the change of direction on young soccer players' performance

**DOI:** 10.1038/s41598-022-16558-9

**Published:** 2022-07-18

**Authors:** Moisés Falces-Prieto, Francisco Tomás González-Fernández, Gabriel García-Delgado, Rui Silva, Hadi Nobari, Filipe Manuel Clemente

**Affiliations:** 1 Research Center High Performance Soccer, Marcet Academy, 08035 Barcelona, Spain; 2 SER Research Group, Pontifical University of Comillas, CESAG, 07013 Palma, Spain; 3 Department of Physical Activity and Sport Sciences, Pontifical University of Comillas, CESAG, 07013 Palma, Spain; 4 Escola Superior Desporto E Lazer, Instituto Politécnico de Viana Do Castelo, Rua Escola Industrial E Comercial de Nun’Álvares, 4900-347 Viana do Castelo, Portugal; 5 Research Center in Sports Performance, Recreation, Innovation and Technology (SPRINT), 4960-320 Melgaço, Portugal; 6 Faculty of Sport Sciences, University of Extremadura, 10003 Cáceres, Spain; 7 Department of Motor Performance, Faculty of Physical Education and Mountain Sports, Transilvania University of Braşov, 500068 Braşov, Romania; 8 Instituto de Telecomunicações, Delegação da Covilhã, 1049-001 Lisboa, Portugal; 9 Department of Exercise Physiology, Faculty of Educational Sciences and Psychology, University of Mohaghegh Ardabili, Ardabil, 56199-11367 Iran; 10 Sepahan Football Club, Isfahan, Iran

**Keywords:** Physiology, Engineering

## Abstract

The aim of the present paper was to determine the relationship between linear sprinting and jump performance, dynamic balance and change of direction on young soccer players. Ninety-four healthy young highly trained male soccer players belonging to the same high-performance academy agreed to participate in the study [twenty-seven soccer players U16 (14.8 ± 0.4 years; height: 170.6 ± 5.6 cm; body mass 64.7 ± 8.4 kg)] and [sixty-seven soccer players U19 (16.6 ± 1.3 years; height: 173.7 ± 7.2 cm; body mass 66.7 ± 8.0 kg)]. Participants completed 3 testing sessions, 7 days apart. Data from a CMJ, Crossover Hop Test, 10-m sprint test, 505 COD tests and the 90° COD test were collected. Moderate correlations were found in some of the cases (r values were between 0.2 and 0.5 in all cases, being *p* < 0.05), indicating that linear sprinting, jumping performance and dynamic balance are influential factors in agility but are not the main limiting factor. The highest correlation was found between the cross-over hop test and the 505 COD test (r = 0.44; *p* < 0.001). The main evidence from the current study suggested that linear sprinting, jumping performance and dynamics balance are determinants of COD, namely explaining the variations in such a skill. The current study revealed that short-distance sprint and jumping performance significantly explain the variations of COD performance on young soccer players.

## Introduction

Multidirectional-based team sports such as soccer, involves a great amount of high-intensity and explosive actions, mainly comprised of linear sprints, jumps and changes of direction (COD) which are part of some of the most required physical capacities to outperform the opponents during competition^1^. Also, soccer players are required to perform both accelerations and decelerations with COD involved in-between. These high-intensity actions, such as accelerations, decelerations and sprints, usually starts with players moving at low running speeds or even from a stopped position^2^.

An important concept that must be emphasized is the reductionist nature of COD ability as a component of player’s agility. While agility is currently defined as an “open-skill” involving unplanned action-reaction tasks, COD tasks are defined as an “closed-skill” involving pre-planned actions^3,4^. Although it is important to distinguish COD from agility, as these two seem to be relatively independent dimensions, it is also imperative to acknowledge the issue of using COD tests in the name of agility performance^5^. That is, to assess agility performance, only tests involving unplanned action-reaction tasks can be conducted, while to assess COD performance, tests involving pre-planned actions must be conducted^5^.

One can argue that pre-planned COD tasks are not as common to happen during a soccer match as it is for baseball, where the players know exactly the COD tasks they have to perform^5^. However, in some moments of the soccer game, such as during set pieces, the team’s strategy may involve pre-planned actions in which COD tasks can be critical for scoring a goal^6^. Furthermore, COD tasks can be a complex and multifactorial skill that may be dependent on each player’s physical characteristics and capacities^7^. In fact, there is a growing interest on the physical determinants that may influence athletes' performance during COD tasks^8–10^^.^

Indeed, interesting correlations between 15 and 30-m sprint performance and COD performance at different angles were previously documented^11,12^. A recent study also found moderate to large relationships between short sprint (10-m) performance, countermovement jump (CMJ) performance and COD performance at different angles (180° or 90°)^13^. However, the same authors stated that despite such correlations were found, both linear sprint and jump performance seem to be independent from COD performance as the R-squared values of those correlations varied between 14 and 34%^13^. Also, another study stated that young soccer players with greater maximal speed values may present a lower performance in COD tasks than their slower counterparts^14^. This fact can be explained by the difficulty that faster athletes present in braking after an acceleration and/or a sprint^9^.

Furthermore, dynamic balance tests are useful to detect possible lower-limb asymmetries that can cause greater risks of injury occurrence, especially in team sports context, as there are high volumes of unilateral-based movements^15^. It was recently showed that dynamic balance performance was significantly associated with COD performance^16^. In addition, also was found that asymmetries in dribbling and change of direction performance were not in agreement to favor the same direction, also displaying a significant difference to each other^17^. However, Rouissi et al., 2018, revealed that the contribution of dynamic balance on COD performance was angle dependent^16^. Given that, the above-mentioned findings reinforce the need to assess and prescribe individualized training programs to enhance sprint, jump and COD performance.

Although extensive literature is available regarding the associations between sprint, jump and COD performance, there is still a lack of consistency regarding the relationships between COD and jump performance. In fact, some studies reported correlations with statistical significance between both physical dimensions^18,19^, while others reported unclear correlations^20,21^. However, a few studies above-mentioned studies were conducted on youth soccer^22,23^. Although there is a growing interest on the relationships between COD performance and the lower-limb asymmetries observed during unilateral jump performance, inconsistent findings were previously found^24,25^.

Given the above-mentioned inconsistencies regarding such associations, especially in youth soccer settings, the aim of the present paper was to determine the relationship between linear sprinting and jump performance, dynamic balance and change of direction. In this sense, we hypothesized that linear sprinting, jumping performance and dynamics balance are crucial of COD performance.

## Material and methods

### Participants

Ninety-four elite young highly trained male soccer players belonging to the same high-performance academy agreed to participate in the study with at least 3 years of competitive experience in regional category and that they stayed a full season at the academy. All participants were familiar with the evaluations carried out. Furthermore completed 9 h of soccer training plus 1 competitive match per week. All parents and participants were informed about the purpose of the study and signed an informed consent before the start of the study, detailing the possible benefits and risks of the research. In this sense, the inclusion criteria were as follows i) previous experience of ≥ 5 years ii) Participants who had no suffered some type of traumatic, muscle–tendon, or neural injury in the 3 months prior to performing the tests were excluded from the study; iii) belong in the academy a full season; iv) participating in ≥ 80% of training sessions, since July to April 2020/2021, and v) participating in all tests proposed and v) giving consent.

The groups were assigned into two categories under 16 (U16) [(n = 27); age: 14.8 ± 0.4 years; height: 170.6 ± 5.6 cm; body mass: 64.7 ± 8.4 kg)] or under 19 (U19) [(n = 67); age: 16.6 ± 1.3 years; height: 173.7 ± 7.2 cm; body mass: 66.7 ± 8.0 kg)]. Concerning the sample size, the following equation was used: *Sample Size* = *Z*2 × (*p*) × (1 − *p*)/*C2*, where Z = confidence level (95%); *p* = 0.05 and *C* = margin of error 0.05. The participants were treated according to American Psychological Association (APA) guidelines, which ensured the anonymity of participants’ responses. In addition, the study was conducted in accordance with the ethical principles of the Helsinki declaration for human research and was approved by the Research Ethics Committee of the Pontifical University of Comillas (internal project no: 2021/65).

### Experimental design

**Figure 1:**
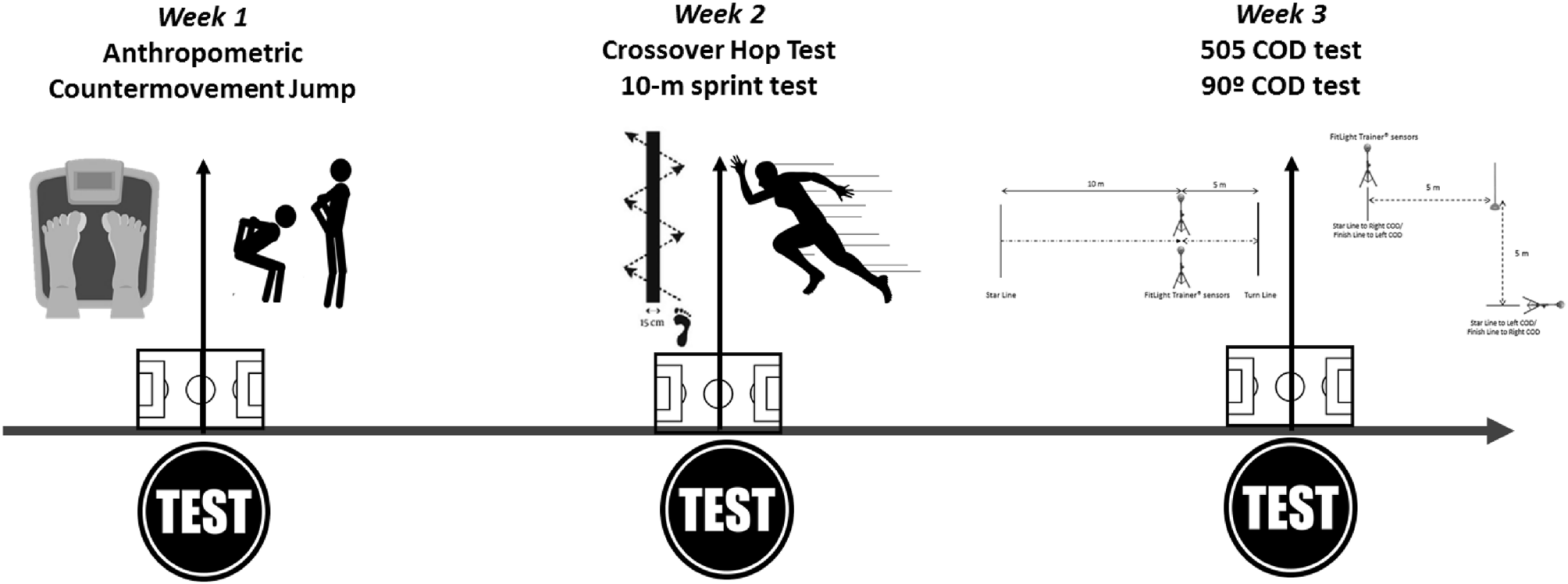
Schematic representation of a test day (see text for full description).

Participants completed three testing sessions, 7 days apart, as you can see in Fig. 1. During each session, data from a CMJ, Crossover Hop Test, 10-m sprint test, 505 COD tests and the 90° COD test were collected. During testing session 1, anthropometric data also were measured and including. Participants were familiar with all the tests the from their regular fitness testing battery. To account for circadian variability, both testing sessions were completed at the same time of day and during participants’ regular training times. This study was conducted in April of 2020/2021 season on day-4 (Wednesday), allowing a rest of 72 h prior to match and within the usual training time (15:30–18:00 h). The assessments were carried under weather conditions (29 °C and 50% humidity). The tests were carried out on a 3rd generation synthetic grass playing surface with a height between 35 and 70 mm and with a distance between them that allows the incorporation of a padding.

### Procedure

Prior to conducting any tests, participants conducted a standardised warm up considering the protocols of Fletcher and Monte-Colombo^26^. Thus, they completed an aerobic activity (continuous run), dynamic stretching, progressive sprinting, and submaximal pre-planned changes of direction, lasting 10 min, the soccer players are familiarized with all exercise. Following the standardised warm up, participants received verbal instruction and demonstrations from the research team immediately prior to conducting 2 familiarisation attempts for each test. Recovery intervals between attempts were standardised at three minutes for each test. For the selection of the dominant leg, the players were asked which leg they preferably use to control, pass and throw the ball regardless of playing position^27^. All the evaluations were performed in the same time and space, before to training session, with the usual clothing for the soccer player, the specific footwear and supervised by the same technical specialists.

### Variables measured

#### Anthropometric characteristics

Anthropometric measurements were taken before the physical testing. First, body composition (BC) was evaluated in the morning (8:00 am) during the first evaluation day without breakfast and wore only shorts and removed any metal and jewelry prior to assessment^28^. For the evaluation of BC, the Bioelectrical Impedance Analysis (BIA) method was used with a TANITA^®^ (MC-980MA PLUS, Arlington Heights, Illinois). BIA is a widely used method for estimating LM and offers a method economic and non-invasively assess the fluid distribution and BC of young soccer players. Secondly, the stature was measured with a stadiometer (Seca^®^ 206, Hamburg, Germany).

#### Countermovement jump

The CMJ was evaluated using the Chronojump-Boscosystem^®^ (Barcelona, Spain) that presents an intraclass correlation between 0.821 and 0.949 to measure the high jump^29^. This system was connected to a MacBook Pro (macOS Sur 11.1). The values were analyzed with a chronopic and recorded by Chronojump version 2.0.2. After a warm-up, participants performed the CMJ test three times on a contact platform with every load jump, with 20 s (sec) of recovery between attempts to minimize the effect of fatigue and three minutes between the different load jumps^30^. The best jump in centimeters (cm) was considered as the final outcome. They were instructed to jump as high as possible after reaching a knee angle of ~ 90°. Participants were also instructed to keep their hands on the hips during the CMJ and to land with their legs extended with maximal feet plantar flexion. If any of these requirements were not met, the trial was repeated.

#### Crossover hop test

**Figure 2:**
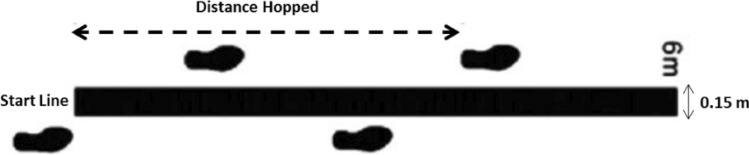
Set up for the crossover hop test.

For the crossover hop test, the participant performed three consecutive jumps over a 15-cm line that had been marked on the floor (Fig. 2). The test consists of performing three jumps in monopodal support and landing with the same leg with which your impulse^31^. Subjects were instructed to place their hands on their hips and to maintain the landing position for 3 s, without loss of balance or performing additional movements involving the free limb. The distance reached was measured in cm from the take-off line to the heel in the final position^32^. They made two attempts and the best was selected.

#### 10-m sprint test

Three sprint test (10 m) were performed with 2 min of recovery between sprints. The times of each sprint were taken in sec. The evaluation system was carried out through FitLight Trainer^®^ sensors (Ontario, Canada). Timing gates were adjusted to an appropriate hip height as per the mean stature of the sample group. The recorded time for each of the players was stored in a portable tablet with an Android system and its subsequent analysis in the Microsoft Windows^®^ Excel program (Redmond, Washington, USA). For data analysis, the average of the three attempts made in each series for subsequent analysis. For the evaluation, 2 Led sensors were placed on a bar 1 m high and in a straight line at distances 10 m. At the light signal, the player sprinted in a straight line down an 80-cm lane from the sensor.

#### 505 COD test

**Figure 3:**
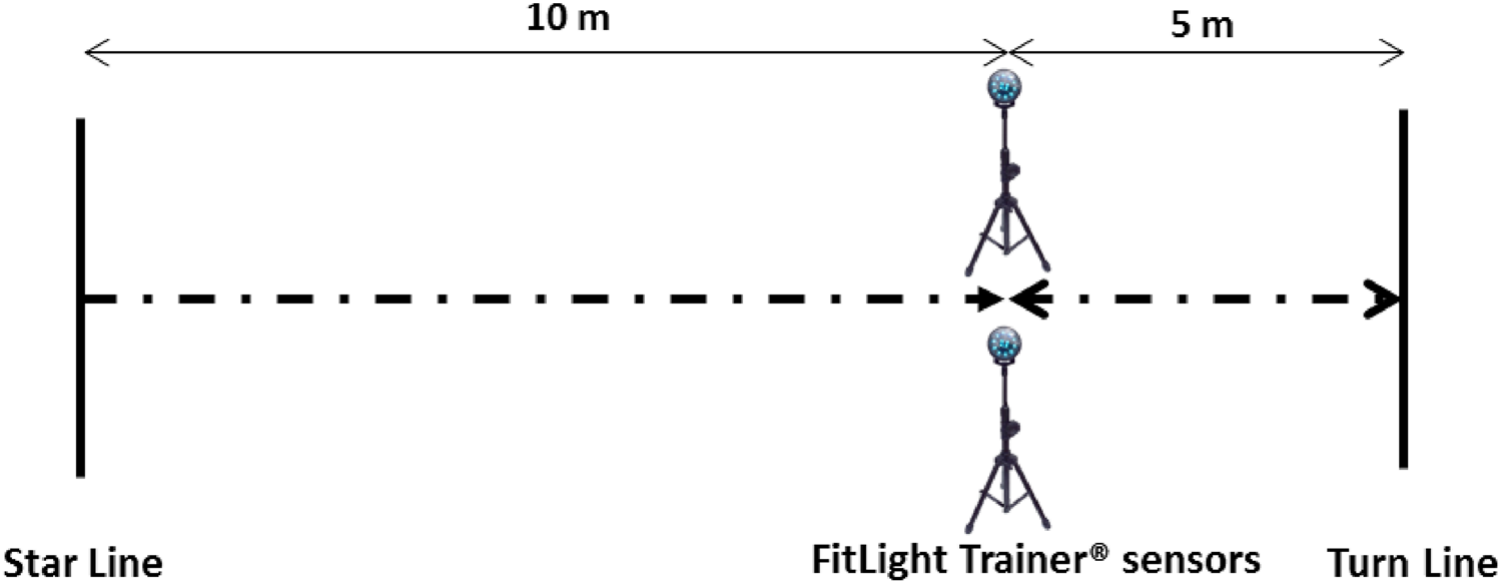
Set up for the 505 COD test.

The methodology for the 505-COD was as per originally established methods^33^, see Fig. 3. Therefore, this involved a 10-m linear sprint from a static start, a 180° turn on a predetermined turn leg (right/left) ensuring contact with a designated line, and a 5-m return sprint through an identified finish line. The time taken to complete the final 5 m of the 10-m linear sprint, turn, and 5 m return sprint was recorded^34^. For speed evaluation, 2 attempts were performed with a recovery time of 2 min between repetitions and an average of the two repetitions for subsequent analysis. Times were measured in sec. As happened in 10-m sprint test, the evaluation system was carried out through FitLight Trainer^®^ sensors. Timing gates were adjusted to an appropriate hip height as per the mean stature of the sample group. The recorded time for each of the players was stored in a portable tablet with an Android system and its subsequent analysis in the Microsoft Windows^®^ Excel program (Redmond, Washington, USA).

#### 90° COD test

**Figure 4:**
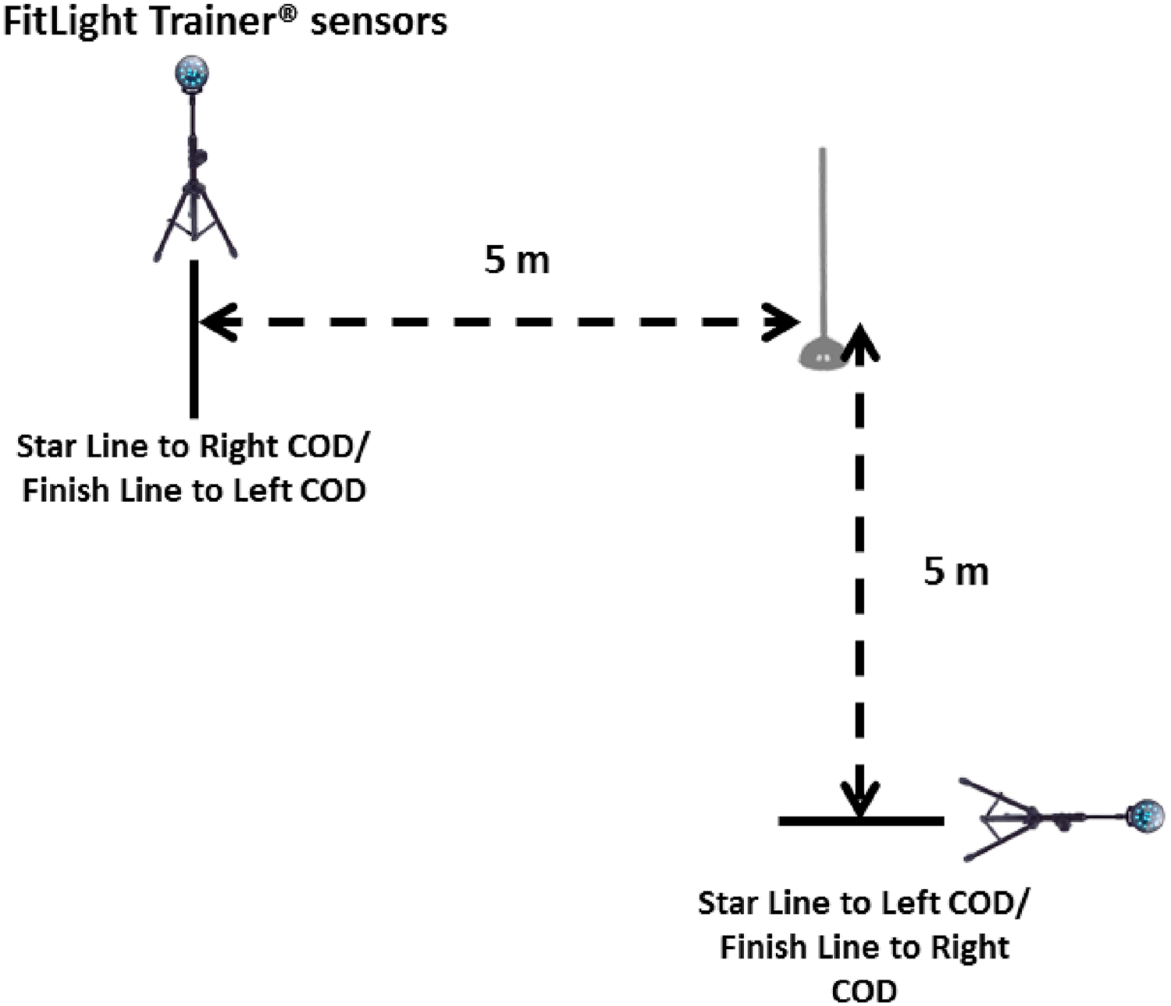
Set up for the 90° COD test.

Finally, they performed three 90° COD test (right/left) (10 m) with 90° (COD) m, see Fig. 4. The times of each repetition were taken in sec. For data analysis, the average of the two attempts made in each series with 2 min of recovery amongs them was chosen. For the evaluation, 2 Led FitLight Trainer^®^ sensors were placed, one at the beginning and the other at the end of the route. Timing gates were adjusted to an appropriate hip height as per the mean stature of the sample group.

### Statistical analysis

Mean and standard deviation were calculated for each variable. For descriptive purposes, partial scatter plots were also calculated between CMJ, Crossover Hop Test, 10-m sprint test and the change of direction tests (505 COD tests and the 90° COD test). Before any parametric statistical analyses were performed, the assumption of normality was tested with the Kolmogorov–Smirnov test on each variable (the results of this test are included in supplementary table 1). The relationship between the result of the jump tests (CMJ), dynamic balance (cross-over hop test) and speed (10 m sprint) and the result of the agility tests (505 test and 90° COD test) was analyzed with multiple linear regressions, including each of the tests and the age category as independent variable (regression p-values and adjusted R-values were calculated). In addition, a multiple linear regression equation was calculated, including the results of the jump height in the CMJ, the time in the dynamic balance test, the time in the 10-m sprint (as continuous) and the age category (as categorical) as independent variables and the result in the change of direction test as a dependent variable. The inflation factors of the variance were computed to verify that the collinearity was not a serious concern.

Statistical analysis was performed with Origin Lab software (based on the tool *General Linear Regression* that allow to include continuous and categorical variables) and the significant p value for the regressions was set at 0.05. The effect size was evaluated using the Evan’s scale^35^: i) 0–0.019, very weak; ii) ≤ 0.20–0.39, weak; iii) ≤ 0.40–0.59, moderate; iv) ≤ 0.60–0.79, strong, and v) ≤ 0.80–1.00, very strong.

## Results

The mean and standard deviation for the test were of 35.5 ± 5.1 cm for the CMJ, of 5 ± 0.7 m for the cross-over hop test, of 2 ± 0.2 cm for the 10 m sprint of 2.4 ± 0.2 s for the test 505 COD test and of 2.7 ± 0.2 s for the 90° COD test.

**Figure 5:**
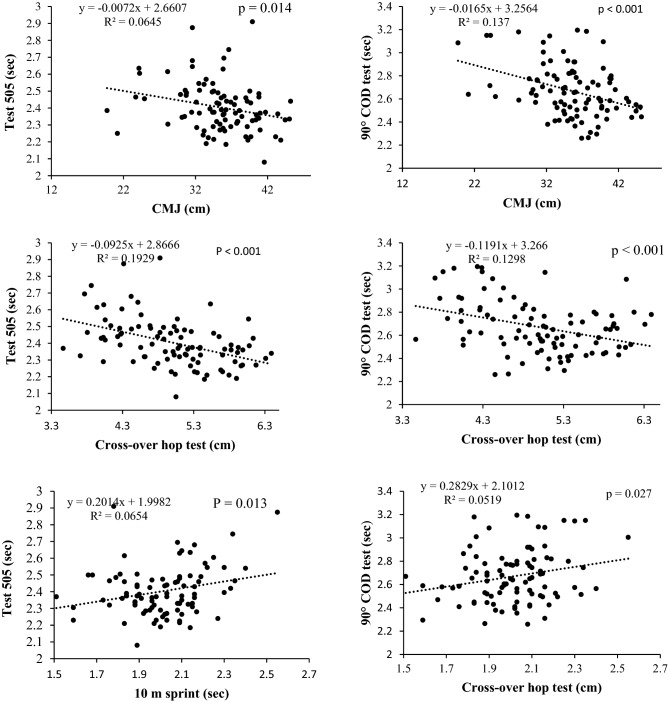
Partial regressions between the jump test (CMJ), the dynamic balance test (cross-over hop test), the sprint test (10 m) and the change of direction test performed (505 test and 90° COD test).

Significant correlations (p < 0.05) were found between the jumping test (CMJ), the dynamic balance test (cross-over hop test), the speed test (10 m sprint) and the result in the two direction change tests performed (including the age category as independent variable in the regression model). According to the Evans scale correlations were moderate in all 6 regression models (Table 1). The highest correlation was found between the cross-over hop test and the 505 COD test (adjusted R = 0.56; Table 1; Fig. 5). There were significant differences by age category in all cases (Table 1).

**Table 1:** Summary of the 6 multiple linear models carried out (including the physical test and the age category as independent variable and the change of direction test as dependent variable).

Model no	Continuous independent variable	Categorical independent variable	Dependent variable	*P* value (age category)	*P* value (model)	R adjusted
1	CMJ (cm)	Age category (U16/U19)	505 test (s)	< 0.001	< 0.001	0.47
2	10 m sprint test (s)			< 0.001	< 0.001	0.5
3	Crossover hop test (s)			< 0.001	< 0.001	0.56
4	CMJ (cm)		90° COD test (s)	0.014	< 0.001	0.41
5	10 m sprint test (s)			< 0.001	< 0.001	0.40
6	Crossover hop test (s)			0.002	< 0.001	0.44

The adjusted R value of the regression that included CMJ jump height, time in the cross-over hop test, time in the 10 m sprint and age category as independent variables and time in the 505 COD test change of direction test as the dependent variable was 0.55 (Table 2). When the dependent variable was time in the 90° COD test the adjusted R value was also 0.46 (Table 3).

**Table 2:** Multiple linear regression (dependent variable: 505 test time; independent variable: countermovement jump (CMJ) height, cross-over hop test (CHT) distance; 10 m sprint time (sec) and age category (U16/U19).

	Coeff	Std err	t stat	*p* value	Lower	Upper	Vif
Intercept	2.6	0.21	12.31	0	2.18	3.01	
CMJ (cm)	0	0	− 0.01	0.99	− 0.01	0.01	1.33
CHT (cm)	− 0.06	0.02	− 3.19	0	− 0.1	− 0.02	1.14
10 m sprint (sec)	0.1	0.07	1.46	0.15	− 0.04	0.24	1.07
Age category (C/J)	− 0.12	0.03	− 3.6	0	− 0.19	− 0.05	1.44

Regression *p* value < 0.001; Adjusted R = 0.55.

**Table 3:** Multiple linear regression (dependent variable: 90° COD test time); independent variable: countermovement jump (CMJ) height, cross-over hop test (CHT) distance; 10 m sprint time (sec) and age category (U16/U19).

	Coeff	Std err	t stat	*p* value	Lower	Upper	Vif
Intercept	3.2	0.35	9.15	0	2.5	3.89	
CMJ (cm)	− 0.01	0	− 2.08	0.04	− 0.02	0	1.35
CHT (cm)	− 0.08	0.03	− 2.45	0.02	− 0.14	− 0.02	1.15
10 m sprint (sec)	0.14	0.12	1.2	0.24	− 0.09	0.37	1.07
Age category (C/J)	− 0.09	0.05	− 1.66	0.1	− 0.2	0.02	1.46

Regression *p* value < 0.001; Adjusted R = 0.48.

## Discussion

The aim of this study was to test the relationships between COD and linear sprinting, jumping performance and dynamic balance. Moderate correlations were found in some of the cases, indicating that linear sprinting, jumping performance and dynamic balance are influential factors in agility but are not the main limiting factor. The highest correlation was found between the cross-over hop test and the 505 COD test. The main evidence from the current study suggested that linear sprinting and jumping performance are determinants of COD, namely explaining the variations in such a skill. There were also differences in all physical test between U16 players and U19 players.

Changing direction represents a combination of different factors. Linear sprinting speed may influence COD, while angle and entry velocity can also constraint the COD performance^36^. Additionally, body mass, lower limb power, and strength can also play important roles to dictates the COD performance^37^. In the current study, it was confirmed that jumping performance and linear speed in 10-m test were determinants of COD performance. Such evidence is in line with previous studies conducted in different sports^37–40^.

To change of direction, it is necessary an acceleration and propulsion phase which requires ground contact times longer than 250 ms large angular displacement between the joints thus it is expectable that long stretch shortening cycle play a more determinant role in COD^41^. Since CMJ is an example of long stretch shortening cycle action (~ 500 ms) requiring time to produce force to propulsion^42^, it would be expectable strong associations between COD and CMJ. Thus, lower limb power (represented by CMJ and cross-hop) can be considered a determinant of COD^43^, namely in the acceleration phase in which a lot of force must be produced quickly to overcome the inertia in both vertical orientation (i.e., CMJ) and horizontal orientation (i.e., cross-hop)^8^.

Another determinant found in this study was linear speed (short distance) which is line with some evidence^8,13^. Linear speed at 10-m is predominantly associated with acceleration, and considering that acceleration is one of the key components of COD can be partially explained the associations between COD and linear speed at short distance. Although being faster in acceleration may not be an overall predictor of COD performance (since players faster are typically worse in braking phase)^43^, a proportion of COD performance is dependent from maximum acceleration, thus being in relation with the linear sprint performance.

Although the findings reported, this does not mean that being stronger and faster can directly improve COD skills, since COD drills should be implemented to a neural activation while taking advantage of the gains of speed and power to finally improve COD performance^14^. Therefore, as practical implications it is important to suggest not only maximize the strength, power and speed of soccer players for improving COD performance, but also apply COD exercises to improve the transference.

### Limitations of the study

As any study, the current research has some limitations. Since correlations are test-dependent, it is expectable that other COD test and other lower-limb power measures, or linear speed distance may produce different relationships. Moreover, other important factors as body composition, body mass and maximal strength or eccentric strength were not tested, thus not being possible to produce a complete model to explain COD performance. Finally, another limitation is the fact of an intermediate age as the under-17 has not been included which does not allow us to analyze the continuum of physical qualities relationships across these ages. Despite the limitations, this study confirms the importance of jumping and speed to explain the COD performance.

## Conclusion

The current study revealed that short-distance sprint and jumping performance significantly explain the variations of COD performance on young soccer players. Although study limitations, the evidence is in connection with past studies, suggesting that speed and power are important factors for improving COD speed. This should be considered by coaches in the moment of prioritizing the training process for improving COD. Future studies may consider analyzing the variations of such relationships across the season, and combine other measures to improve the regression model.

## Supplementary Information

Supplementary Information.

## Data Availability

The datasets generated during and analyzed during the current study are available from the corresponding author on reasonable request.

## Abbreviations

CMJ: Countermovement jump
COD: Changes of direction
APA: American Psychological Association
BC: Body composition
BIA: Bioelectrical impedance analysis
