# Supplementary material for: Relationship between sprint, jump, dynamic balance with the change of direction on young soccer players' performance
Source: Sci Rep. 2022 Jul 18;12:12272. doi: 10.1038/s41598-022-16558-9 (PMC9293905; doi:10.1038/s41598-022-16558-9)
Supplement: Supplementary file 1 — Supplementary Information. [file 41598_2022_16558_MOESM1_ESM.docx]

| **Supplementary table 1.** Kolmogorov Smirnov normality test by physical test and age category | | | | | |
| --- | --- | --- | --- | --- | --- |
| Test | Group | DF | Statistic | p-value | Decision at level (5%) |
| CMJ (cm) | Under 16 | 27 | 0.94 | 0.17 | Can't reject normality |
|  | Under 19 | 67 | 0.97 | 0.23 | Can't reject normality |
| Crossover hop test (cm) | Under 16 | 27 | 0.97 | 0.64 | Can't reject normality |
|  | Under 19 | 67 | 0.98 | 0.35 | Can't reject normality |
| 10-m sprint test (s) | Under 16 | 27 | 0.98 | 0.96 | Can't reject normality |
|  | Under 19 | 67 | 0.98 | 0.62 | Can't reject normality |
| 505 COD test (s) | Under 16 | 27 | 0.94 | 0.20 | Can't reject normality |
|  | Under 19 | 67 | 0.98 | 0.42 | Can't reject normality |
| 90º COD test (s) | Under 16 | 27 | 0.12 | 0.82 | Can't reject normality |
|  | Under 19 | 67 | 0.11 | 0.29 | Can't reject normality |
